# Endoscopic mucosal resection of a bleeding gastric carcinoid in a resource-limited setting: a case report

**DOI:** 10.1093/omcr/omaf203

**Published:** 2025-10-29

**Authors:** Salma Barakat, Rayan Yousif, Ahmed Rafei, Rawan A Bedab, Abdelmounem E Abdo

**Affiliations:** Department of Hepatology, National Center for Gastrointestinal and Liver Diseases, Mohammed Najeeb Road, Ibn Sina Specialized Hospital, Khartoum State, 1500, Sudan; Research Department, National Center for Gastrointestinal and Liver Diseases, Mohammed Najeeb Road, Ibn Sina Specialized Hospital, Khartoum State, 15004, Sudan; Research Department, National Center for Gastrointestinal and Liver Diseases, Mohammed Najeeb Road, Ibn Sina Specialized Hospital, Khartoum State, 15004, Sudan; Research Department, National Center for Gastrointestinal and Liver Diseases, Mohammed Najeeb Road, Ibn Sina Specialized Hospital, Khartoum State, 15004, Sudan; General Director, National Center for Gastrointestinal and Liver Diseases, Mohammed Najeeb Road, Ibn Sina Specialized Hospital, Khartoum State, 15004, Sudan

**Keywords:** gastric carcinoid, neuroendocrine tumor, hematemesis, endoscopic mucosal resection, endoscopic ultrasound, minimally invasive treatment

## Abstract

Gastric carcinoids (GCDs) are rare neuroendocrine tumors that can occasionally present with upper gastrointestinal bleeding. We report a case of a 60-year-old male who presented with hematemesis due to a gastric carcinoid tumor. Emergency gastroscopy identified a bleeding lesion in the lesser curvature, successfully managed with adrenaline injection. Endoscopic ultrasound confirmed a mucosal-confined lesion, and endoscopic mucosal resection (EMR) was performed. Histopathology confirmed a well-differentiated neuroendocrine tumor with negative margins. The patient remained stable post-procedure and was discharged with a planned follow-up. This case highlights the role of EMR as a minimally invasive and effective therapeutic option for bleeding gastric carcinoids, particularly in resource-limited settings. Early recognition and intervention can optimize patient outcomes while avoiding unnecessary surgical procedures.

## Introduction

Gastric carcinoids (GCDs) are rare neuroendocrine tumours (NETs) that originate from enterochromaffin-like (ECL) cells [1]. GCDs can arise sporadically or result from long-term hypergastrinaemia. Due to their rarity—accounting for just 2% of all carcinoids and 8.7% of gastrointestinal carcinoids— researchers have not extensively studied the factors that predict metastatic potential [2, 3]. Most patients with gastric carcinoid tumours (GCTs) are middle-aged females [4].

Occasionally, GCDs can lead to complications such as upper gastrointestinal bleeding. This is particularly relevant in understudied regions like Africa and Sudan, where there are few documented cases. Although endoscopic mucosal resection (EMR) is a minimally invasive procedure, its role as a treatment for gastric carcinoids has not been thoroughly investigated [5]. Historically, clinicians considered total gastrectomy the standard treatment. However, recent insights into the varied biological behaviour of these tumours have led to an increased therapeutic use of EMR [6].

In this case report, we present the first documented case in Sudan of a 60-year-old male who developed haematemesis from a gastric carcinoid. This case highlights the need for less invasive treatment approaches.

## Case presentation

A 60-year-old male presented to our emergency bleeding centre with a sudden onset of haematemesis, having lost approximately 0.5 litres of fresh, bright red blood. Upon arrival, he was haemodynamically unstable, with a blood pressure of 85/50 mmHg, a heart rate of 118 beats per minute, and a respiratory rate of 22 breaths per minute. He reported no previous history of gastrointestinal symptoms and had no significant past medical history or prior surgical procedures. A clinical examination revealed no abnormalities.

Initial laboratory investigations revealed a haemoglobin level of 8.1 g/dL, with a normal white cell count and platelet count. Liver function tests—including AST, ALT, bilirubin, and albumin—were within normal limits. Renal function was also normal. Coagulation parameters (PT, INR, and aPTT) were unremarkable. Tumour markers, including chromogranin A and serum gastrin, could not be assessed due to unavailability.

We admitted the patient, adequately resuscitated him, and administered one unit of blood transfusion. After he achieved haemodynamic stability, an emergency gastroscopy revealed a raised lesion in the lesser curvature of the stomach with a spurting vessel. Due to the unavailability of endoscopic clips and Argon Plasma Coagulation (APC) at the time, we successfully controlled the bleeding by injecting adrenaline (1:1000). Following the procedure, we closely monitored the patient’s vital signs.

Once stabilised, the patient was referred for an Endoscopic Ultrasound (EUS) to characterise the lesion, assess its depth, and rule out the possibility of an ectopic pancreas. The EUS, performed with an Olympus radial scope, revealed a well-defined hypoechoic lesion confined to the mucosa with an intact submucosa. No ductal structures were identified, making ectopic pancreas unlikely.

**Figure 1:**
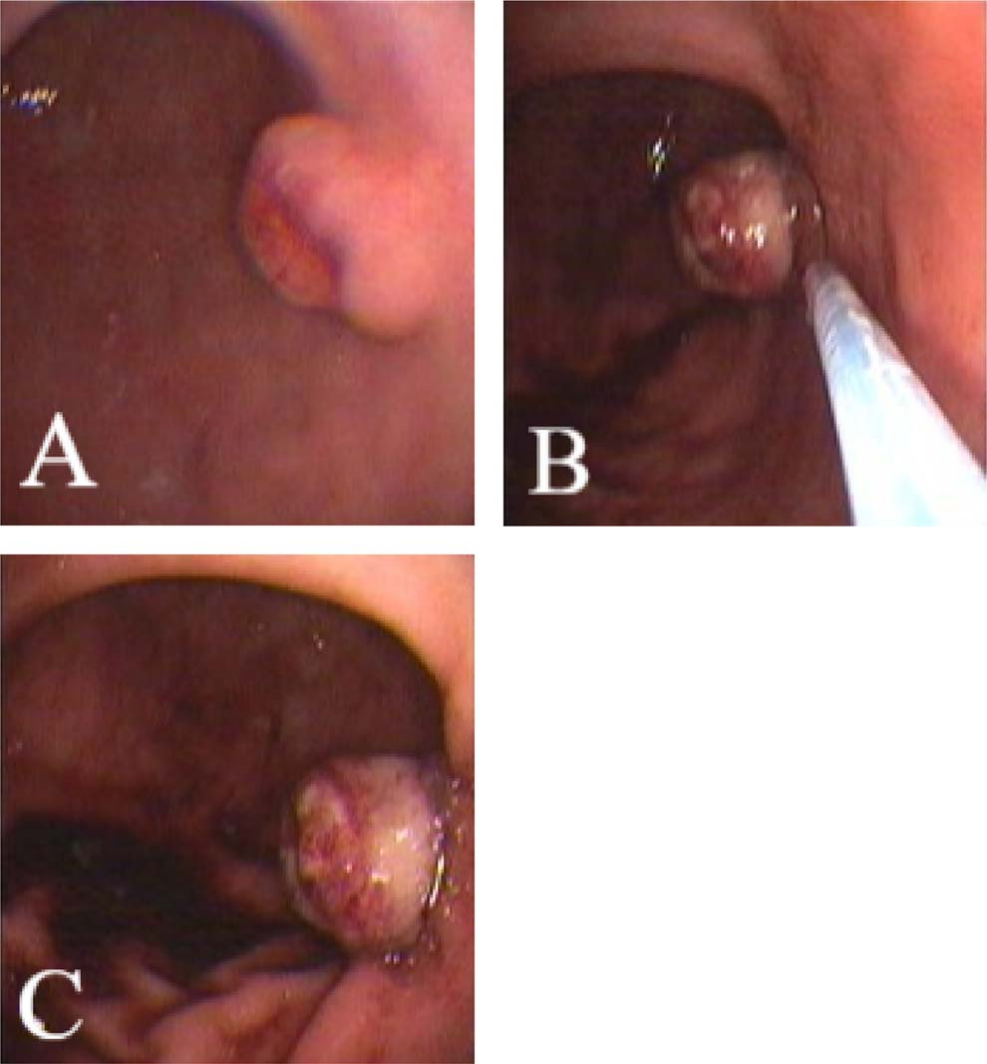
Illustrates the key steps of the EMR procedure: (**A**) the initial endoscopic view of the gastric lesion, (**B**) suctioning of the lesion into the band ligation barrel, and (**C**) the formation of a polyp-like structure following band application.

The patient subsequently underwent EMR using a Pentax EG-2970K endoscope, a band ligation device, and an Acue snare from Wilson-Cook Medical. Initially, the lesion was examined (Fig. 1A), and the endoscope was withdrawn. The band ligation barrel was then inserted, and the endoscope reintroduced. Using suction, the lesion was drawn into the barrel and a band applied (Fig. 1B), creating a polyp-like structure (Fig. 1C).

The endoscope was withdrawn again. Upon re-entry, the captured mucosa was resected using electrocautery with a snare below the band. The specimen was collected using a Dormia basket. No bleeding occurred at the resection site, and the specimen was sent for histopathology.

**Figure 2:**
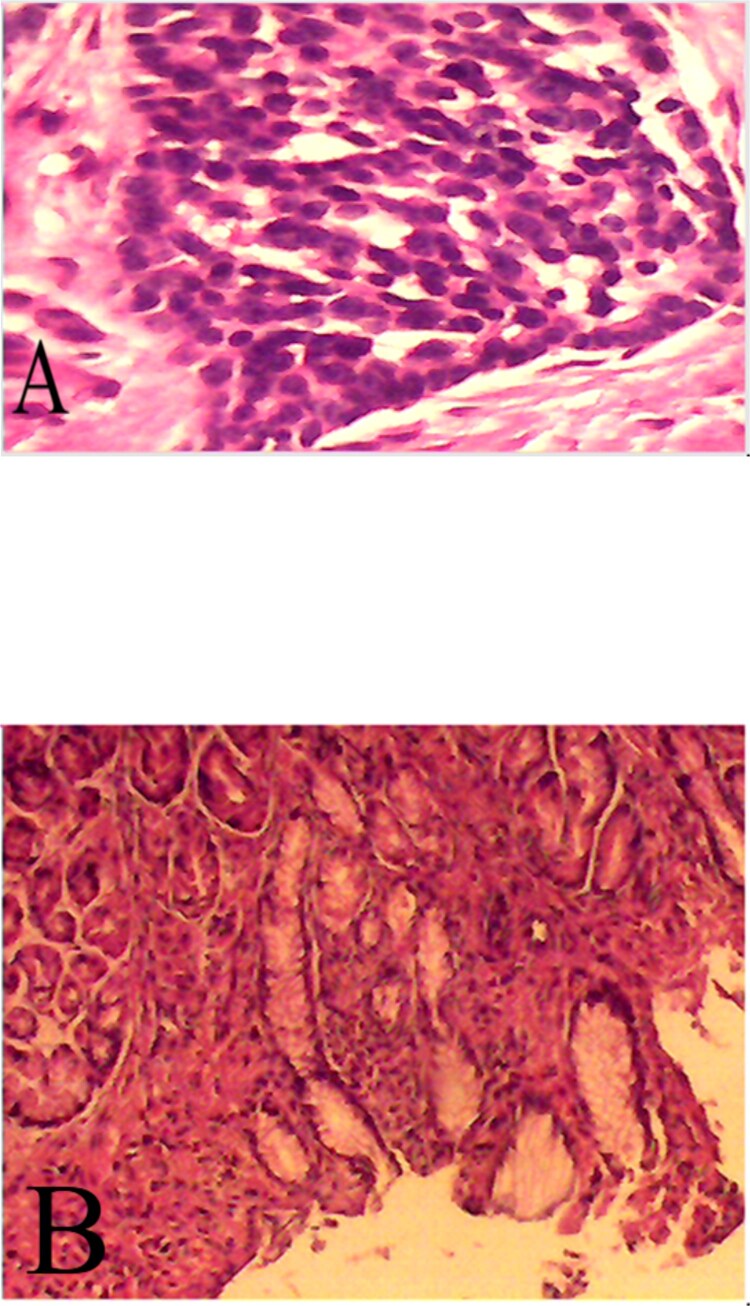
Provides histopathological confirmation of the diagnosis: (**A**) a microscopic view of the well-differentiated neuroendocrine tumor, and (**B**) tumor cells displaying characteristic fusiform morphology with darkly stained, uniform nuclei embedded within fibrous tissue, demonstrating clear resection margins.

Histopathology confirmed a well-differentiated neuroendocrine (carcinoid) tumour (Fig. 2A), composed of rounded fusiform cells with darkly stained, uniform nuclei, surrounded by fibrous tissue with clear margins (Fig. 2B).

The patient remained stable post-procedure and was closely monitored. He improved and was discharged with a follow-up gastroscopy planned in 3 to 6 months.

Post-procedure, a contrast-enhanced CT scan of the abdomen was performed, which showed no evidence of residual tumour or metastatic disease. The patient was referred to the oncology department for ongoing surveillance. His recovery was smooth, with no adverse events or unanticipated outcomes during hospitalization or early follow-up.

## Discussion

This case report adds to the growing evidence supporting EMR as a first-line therapy for mucosal gastric lesions that present with gastrointestinal bleeding. This is particularly relevant in resource-limited settings where there may be a shortage of surgical expertise.

We selected EMR as the treatment of choice because it is minimally invasive and can be a curative approach for small carcinoid tumours [7]. EUS is crucial for determining the appropriate management of polypoid lesions, especially for assessing their depth within the gastrointestinal wall layers. EUS helps to determine if a lesion is confined to the mucosa or submucosa, which directly influences whether it can be safely removed endoscopically or requires surgical intervention [8].

One of the key diagnostic challenges in this case was the initial differentiation of the bleeding source. In the acute setting, common causes of upper gastrointestinal bleeding such as gastric ulcers, bleeding polyps, and vascular lesions like Dieulafoy’s lesions were considered. The raised, subepithelial nature of the lesion observed on gastroscopy prompted further evaluation to exclude entities such as ectopic pancreas or gastrointestinal stromal tumours (GISTs), which can also present with similar appearances and bleeding [9]. The lesion’s resemblance to an ectopic pancreas added further complexity. EUS played a critical role in characterising the lesion, confirming its confinement to the mucosa and ruling out features suggestive of pancreatic tissue, such as ductal structures [8].

Several studies and case reports show that EMR can effectively treat gastric carcinoids, especially those associated with bleeding. Shimoyama et al. described the successful use of EMR to treat a patient with hypergastrinaemia and gastric carcinoids. They noted that the procedure effectively reduced bleeding and completely removed the tumour with minimal complications [10]. Similarly, Lin et al. reported a case of a gastric carcinoid associated with a GIST. EMR successfully treated the non-metastatic carcinoid, emphasising the importance of comprehensive diagnostic imaging and biopsy in guiding treatment [11]. In their study on managing small duodenal carcinoids with EMR, Shroff et al. also reported a high success rate with minimal post-procedure complications [7].

Conversely, while Dakin et al. found EMR effective for well-differentiated, manageable type 1 gastric carcinoids, they suggested that some cases may require additional treatments depending on the tumour characteristics [12]. Spampatti et al. reported an exceptionally aggressive case of a type 1 gastric carcinoid that was successfully treated with EMR, highlighting the importance of careful patient selection and meticulous follow-up [13].

These studies collectively show that EMR is a viable treatment for bleeding gastric carcinoids. In our case, the complete excision of the tumour with negative margins and the absence of post-procedural complications underscore the efficacy and safety of EMR. Future research should focus on the long-term outcomes of patients treated with EMR, particularly concerning recurrence rates and the potential need for adjuvant therapy.

This case highlights the effective use of EUS and EMR in a resource-limited setting. EUS played a key role in establishing an accurate diagnosis, while EMR offered a safe and minimally invasive treatment option. However, our approach was limited by the unavailability of tumour markers such as chromogranin A and serum gastrin, which could have provided additional diagnostic and prognostic information. Despite these constraints, the case was managed successfully, underscoring the importance of clinical judgement and adaptability in low-resource environments.
